# Plague and Quarantine

**Published:** 1898-11-05

**Authors:** 


### Hospital Clinics and Medical Progress.

### PLAGUE AND QUARANTINE.

The remarks which we made last week in regard to the inutility of quarantine as a means of controlling the progress of plague have led some of our readers to protest that, after all, the institution of quarantine is a measure sanctioned by the experience of many ages, that it is the only thing that has ever stood in the way of progress of the disease, and that the natural quarantine imposed by the intervention of long sea voyages has in all ages forced plague (as, indeed, it has other epidemic diseases) to travel by long and circuitous land routes rather than to take comparatively simple short cuts by sea. This is true; and if we are prepared to accept in the name of quarantine such an absolute isolation of one country from another as was imposed in the Middle Ages by the intervention of the ocean, we have no doubt that quarantine might be effectual. It may, however, be doubted whether any countries which have once become accustomed to the free intercommunication which is common in these latter days, and especially countries which are so dependent upon such intercommunication for their daily bread as, for example, England is at the present time, would submit to be left, as the saying is, to "stew in their own juice" for a single week to the extent that would be involved in the sort of quarantine which was imposed in the Middle Ages, and which even then failed to do more than merely put off the evil day.

The attempts which have recently been made in India to prevent the spread of plague by interfering with the movements of those who were suspected of having been exposed to the infection have evidently failed in their purpose, and if it be said that they only failed because they were imperfectly carried out, we would ask those who have followed the reports from Bombay whether it is in the slightest degree probable that even such a partial quarantine as failed there could be applied here. Let us, then, turn to an example of suppressive measures which did seem to succeed, and let us consider what successful quarantine really means, always bearing in mind that the instance we are about to relate had to do with a scattered series of villages, and that its apparent success in such circumstances is but small proof that it would be effectual in crowded cities.

The instance to which we would refer is the last occasion on which plague showed its face in Europe, namely, the outbreak which occurred at Vetlianka on the Volga in 1878. According to Dr. Frank G. Clemow, in the *Indian Medical Gazette*, the mortality in this outbreak was very great in the houses which were attacked, carrying off entire households when it had once gained a foothold in them. Among the measures taken to control the spread of the disease we note that those adopted by the villagers themselves appear to have consisted mainly in an attempt to isolate every house in which a case of plague occurred, and completely cut off all communication between the inmates of this and of adjoining houses. "The crudity and cruelty, however, of a measure which consists in shutting up sick and well together in an infected house and leaving them to their fate, are obvious, and there can be little doubt that it was largely responsible for the fearful mortality in individual households to which reference has already been made." But just as the villagers isolated the infected houses with but little care for the fate of their wretched inhabitants, so the empire, for its own protection, isolated the infected villages and the infected districts, with apparently no care at all for the fate of the communities in question, so only that the infection could be kept within the cordon. The Governor General of Astrakhan issued orders that a cordon of troops should be placed round every village or locality in which a case of plague had occurred, and that all communication between such places and the outside world should be cut off until a period of 42 days had elapsed from the occurrence of the last case. A second cordon of Cossacks was drawn round an area some hundred miles in length and fifteen miles in breadth, including the bed of the Volga and all the villages in which plague had appeared. In this cordon the troops were posted at stations two miles apart, a constant patrol being maintained between adjacent stations. A ten days' quarantine was enforced by this cordon, and lastly, a similar cordon was drawn round the entire governments of Astrakhan and Saratof. According to the English report upon the epidemic, "the hygienic measures carried out were of the most uncompromising character, and were executed with a vigour which must have left its mark upon the villages subjected to them for many years to come," and as details of these "uncompromising measures" we may mention that all houses in which a case of plague had occurred, and all articles of furniture or clothing which had been in any way exposed to infection were destroyed by fire. No doubt a quarantine such as this was and would be fairly effectual in holding back for a time the progress of an epidemic, especially when, as in Russia at the season in question, *i.e.*, during the winter, the people were largely confined to their houses by the weather, and the normal work of the district was to a large extent at a standstill. It is well, however, that we should recognise, on the one hand, what quarantine really means if it is to be effectually applied; and, on the other, should bear in mind that, as we have seen in India, even in the midst of plague-stricken localities, the well fed, the well housed, and the more vigorous members of the ruling race are but rarely attacked.

If plague should come, we have no doubt that it would claim many victims, just as cholera did 50 or 60 years ago. We were then unreformed as to water supply, and it is not too much to say that cholera was the stern teacher by whom we were put on the right track in that respect. Sanitation, however, is many-sided, and consists of much besides the provision of pure water. We can have but little doubt that an attack of plague would pick out many weak spots in our sanitary armour, and that in regard to these also the stern lessons of experience would again lead to new reforms as to over-crowding, as to dirt, and as to ventilation, just as the lessons of cholera led to improvement in our water supplies. But would it not be simpler to use the knowledge we possess, instead of waiting to be driven by force or pestilence?

